# Towards a successful teledance program for youth with cerebral palsy: A mixed-method study with the instructor’s perspective

**DOI:** 10.1177/18758894251324317

**Published:** 2025-04-10

**Authors:** Annie Pouliot-Laforte, Claire Cherrière, Margaux Hebinck, Jessica Tallet, Catherine Donskoff, Louis-Nicolas Veilleux, Martin Lemay, Maxime T Robert

**Affiliations:** 1 Centre de recherche CHU Sainte-Justine, Montréal, Québec, Canada; 2 Département des sciences de l’activité physique, Faculté des sciences, Université du Québec à Montréal (UQAM), Montréal, Québec, Canada; 3 Toulouse NeuroImaging Center (ToNIC), Université de Toulouse Inserm UPS, Toulouse, France; 4 Soins de Suite et de Réadaptation pédiatrique Paul Dottin ASEI, Ramonville St-Agne, France; 5 School of Rehabilitation, Faculty of Medicine, Université Laval, Québec, Québec, Canada; 6 Centre for Interdisciplinary Research in Rehabilitation and Social Integration (Cirris), Québec, Québec, Canada; 7 Shriner's Hospital for Children Canada, Québec, Québec, Canada

**Keywords:** cerebral palsy, dance, telehealth, physical activity, leisure time physical activity

## Abstract

**Purpose:**

Dance is a leisure time physical activity (LTPA) known to improve motor, cognitive, and psychosocial functions in youth with cerebral palsy (CP). Online exercise or tele-programs are promising in overcoming the environmental barriers of accessibility to LTPA. To ensure successful implementation, it is necessary to identify limitations specific to dance in a pediatric population. The aim was to explore the perspectives of the main stakeholders, i.e., dance instructors and youth, to implement such a program.

**Methods:**

In a mixed-method design, feasibility indicators were assessed by participation and retention rates, the Physical Activity Enjoyment Scale (PACES), and the Children's Effort Rating Table (CERT). Semi-structured interviews were conducted before and after the intervention with youth with CP [n = 15] and dance instructors [n = 3]. Interviews were analyzed with an inductive approach.

**Results:**

Participation and retention rates were 86.7% ± 10.7 and 100%, and the PACES and CERT average scores were 91% ± 11 and 3.7 ± 1.3, respectively. Four themes emerged from the interviews: 1) Technology; 2) Pedagogical Approach; 3) Participant's Environment; and 4) Social Relations.

**Conclusion:**

The teledance program is feasible and enjoyable, requiring minimal equipment and travel. However, there is a need to consider and provoke social interaction, to enhance the social and relational dimension of dance.

## Introduction

Cerebral palsy (CP) describes a group of permanent disorders affecting the development of movement and posture, causing activity limitations, which are attributed to non-progressive disturbances that occurred in the developing fetal or infant brain.^1^ The health benefits of having an active lifestyle in youth with CP are well known and numerous. Greater participation in leisure time physical activity could help achieve higher fitness levels, reduce secondary complications such as early gross motor functional loss, and improve autonomy and overall quality of life.^2–4^ Moreover, leisure time physical activity is necessary for the optimal physical, emotional, and psychological development of all children.^5^

Dance, a leisure time physical activity, has been shown to improve motor and cognitive functions of young individuals with CP, as well as to provide psychosocial benefits.^6–10^ Specifically, according to a recent scoping review, dancing activities can lead to significant improvements across domains of the International Classification of Functioning such as balance, strength, mobility, self-confidence, body image, and sense of accomplishment.^6^ In addition, dance provides opportunities to engage in a social activity, a motivational environment, and a form of enjoyable creative expression,^10^ which are key to promoting long-term adherence to leisure time physical activity, participation, and thus an active lifestyle.^5^ Yet, community-based dance intervention can be difficult to access, notably due to environmental barriers such as transportation issues or poorly adapted facilities.^11^ Online exercise, or tele-programs (i.e., virtual service provided at a distance), represents a promising approach to improve the accessibility of leisure time physical activity for children and adolescents with CP.^12^ Online exercise has the potential to increase functional abilities in youth with CP due to the targeted nature of the program.^13^ However, while the potential value of a tele-program involving dance has been suggested, little is known about how young individuals with CP will engage with it and what barriers there may be to its online implementation.

To guide the development and deployment of teledance activities for young individuals with CP, identifying potential limitations is crucial. This approach not only aids in addressing the challenges of engaging youth with CP in lifelong leisure time physical activity but also emphasizes the importance of incorporating perspectives from different key stakeholders to uncover facilitators and barriers to a successful teledance program.^14^ Recognizing the significance of stakeholders’ involvement, the design and evaluation of new programs, especially those incorporating new technologies, benefit from the active participation of both participants and instructors.^15^ As primary stakeholders, dance instructors play a pivotal role in designing, coordinating, and delivering these programs. Their insights are invaluable for identifying implementation barriers and strategies, including resource needs, frequency, duration, and technology-related feedback. To the authors’ knowledge, no studies have evaluated the perspectives of dance instructors in implementing a teledance program for young individuals with CP.

Similarly, participant feedback is essential for understanding their enjoyment, engagement, and the limitations they perceived in teledance programs. Challenges in maintaining motivation have been noted in previous home-based programs due to the lack of physical interaction.^16^ Research on adult populations has underscored the difficulty of virtually replicating the social aspects of dance that contribute to well-being.^17^ These observations underscore the necessity of understanding the perspectives of those directly involved, such as dance instructors and participants, to gain comprehensive insights into user needs and the challenges of implementation and motivation critical for the success of future programs.

Thus, the aim of this study was to explore the perspectives of the main stakeholders, i.e., the dance instructors and the children and adolescents, to implement a teledance program in young individuals with CP.

## Methods

### Study design

A mixed-method convergent parallel design was used for this study.^18^ Feasibility indicators were conducted using participation and retention rates, the Physical Activity Enjoyment Scale (PACES), and the Children's Effort Rating Table (CERT). Interviews were conducted with two groups of stakeholders: young individuals with CP and dance instructors. The study was approved by the Research Ethics Board of Sainte-Justine UHC Research Center and the University of Toulouse Ethics Committee (number 2020-284).

### Participants

A convenience sample of 15 children and adolescents with CP was recruited to participate in the teledance program. Participants’ characteristics are presented in Table 1. Six participants were older than 10 years. Among these older participants, only one individual was male, and he had previous experience in dancing. Two participants had mobility aids. Gross Motor Function Classification System (GMFCS) levels varied from I to IV across the participants. The recruitment took place in two rehabilitation centers in Canada and France. Inclusion criteria were a diagnosis of CP; aged between six and 21 years old; the ability to follow simple instructions; and access to a computer or an electronic tablet connected to the internet. Exclusion criteria were any cardiovascular disease and surgical intervention 12 months prior to the teledance program, as well as any restrictions on physical activity. Children and adolescents with and without dancing experience were welcome to participate in the program as well as those who used assistive devices in their daily life. Participants older than 18 years old or parents signed informed consent, and children and adolescents younger than 18 years old gave their assent to participate via a secured platform.

**Table 1.** Participants’ characteristics.

Participant	Sex	Age	Technical aids for mobility	Dancing experience	City of residence
P01	F	7	none	N	A
P02	F	11	none	Y	A
P03	M	7	none	N	A
P04	F	8	none	Y	A
P05	M	19	none	Y	B
P06	F	18	Walker	Y	B
P07	M	7	none	N	B
P08	F	7	none	N	B
P09	M	10	none	N	A
P10	M	8	none	N	A
P11	F	13	none	N	A
P12	F	6	none	N	A
P13	F	18	Electric wheelchair	Y	B
P14	F	10	none	Y	B
P15	F	12	none	Y	B
	10 F / 5 M	10.7 [4.4]*		8 N / 7 Y	

*Data are presented as mean [SD]. F (female); M (male); Y (Yes); N (No); A (Country A); B (Country B).

Three dancing instructors were recruited through the research team's contact networks to design, coordinate, and deliver the teledance program. The inclusion criteria were having experience in teaching dancing classes for young individuals with motor impairment and being available three times a week during the dance program. The instructors had a range of 2–5 years of experience with teaching dance for youth with motor impairments. The instructors initial formation was 1) school dance teacher, 2) neuropsychologist, and 3) physiotherapist with a specific training in adapted and inclusive dance. All instructors signed informed consent to participate in the study via a secured platform.

### Procedures

The teledance program was delivered to a total of four groups. Two followed the program in the summer and the other two in the fall of 2020. Two were from Canada and two from France. Each had between three and four participants. The teledance program consisted of a 15-h span over five weeks (three times a week, one hour per class). Once a week, the participants from the Canadian and French groups were combined in the same virtual class. This joint class was designed to increase motivation by meeting peers from a different country. A minimum of one dance instructor was provided for every four participants. The small number of participants per group was determined based on the limited visibility displayed on the computer screen and to ensure sufficient individual guidance from the dance instructors.^19^ To ensure standardization in the design of the teledance program across all instructors, each instructor received three hours of training focusing on adapted dance, motor learning principles, and the presentation of clinical symptoms in CP. Each dance class was held with music, except during the opening and closing periods. Each class included an opening time to greet participants (three minutes), a warm-up period (10 min), an exploratory activity to discover dance (10 min), a series of exercises practicing dancing style (10 min), a pause while watching dance videos (five minutes), a collective creation of choreography (15 min), a cooldown period (five minutes), and a closing time (two minutes). With the consent of the participants and their parents, a live performance of the dance choreography was presented to the families and professionals at the rehabilitation centers during the last teledance class.

The overall program was tailored to each child's goal, favorite dances, and favorite music styles. Since there were multiple children in each class, the instructors provided group instructions and offered individualized guidance based on each child's goal (e.g., ensuring that a child aiming to dance with both sides of their body could execute movements with both arms). Moreover, the instructors ensured that the music styles preferred by all participants were represented throughout the program. The complexity of movements corresponding to the dancing styles (i.e., swing, hip-hop, afro, Bollywood and classical) was adjusted to suit the functional levels of each participant. For instance, all dance movements could be performed either standing or sitting, depending on the child's mobility. Dancing styles varied from week to week based on the children's preferences, with progressive exercises focusing on one dancing style conducted over one week (three classes). At the beginning of the following week, another dance style was introduced.

To ensure the physical safety of the participants during the teledance program, instructions were given to the individuals and their parents prior to the first class. These instructions included using a safe and unobstructed space big enough to take a step in each direction, being barefoot to avoid sliding on the floor, ensuring the presence of an adult nearby, keeping a chair close for the child to rest if needed, and bringing a water bottle. The dance instructors confirmed that these instructions were followed at the beginning of each class.

### Interviews

Interviews were conducted prior to and immediately after the dance program. Primary stakeholders were divided into two groups, i.e., dance instructors and young individuals with CP. A total of 12 interviews were conducted. Semi-structured interviews lasting approximately 90 min were conducted to gather dance instructors’ perspectives and experience (n = 4). Semi-structured group discussions lasting 30 min were conducted with young individuals with CP to gather the acceptability and implementation criteria of the teledance program (n = 8).^20^ Each group was determined based on their intervention group and ranged between three and four young individuals with CP. Participants could be accompanied by their parents, if needed. All interviews were conducted via a secured virtual platform, i.e., Microsoft Teams (Microsoft, Redmond, USA) or Zoom (Zoom Video Communications, San José, USA). Audio was recorded with independent recorders, transcribed, and coded to suppress reference to participants’ names. Age, country of residence, and previous dancing experience were collected from all participants. Preferences on dancing style and music as well as individual goals were asked of the participants during the pre-interviews. This information was used to develop and personalized the dance program.

For all interviews, a semi-structured interview guide was developed by the multidisciplinary research team, comprising a physiotherapist and kinesiologists specialized in motor learning, motor development, dance intervention, and technology integration. Themes and questions related to the research question were identified by the research team (e.g., What are the barriers and facilitators to implementing a teledance intervention? What are the positive and negative differences between online and in-person dance?). A first version was developed by A.P-L. and then presented to the research team for intra-team validation.^21^ A total of two semi-structured interview guides were developed, one guiding pre-interview and the second guiding post-interview (see supplementary data A and B). A total of eight open-ended questions were included in the interview guides. Questions related to dance in the interview guide pre-intervention included (a) What are your experiences with dance? (b) Describe how you felt, or how you feel about online exercise; (c) What are you looking for in this teledance program? and (d) Name me some goals you would like to achieve. Questions related to dance in the interview guide post-intervention included (a) How do you feel about the dance program provided online? (b) Tell me your level of satisfaction with the teledance program? and (c) Describe a situation where you would have made changes.

### Indicators

Feasibility indicators were collected throughout the teledance program. The acceptability of the program was quantified by the participation and retention rates.^20^ The participation rate was defined as the presence of each participant to each dancing class on the total number of classes (n = 15). The retention rate was defined as the number of participants who completed the five-week dance program. The PACES, a valid and reliable questionnaire for children, was used to quantify participant satisfaction with the dance program.^22^ This scale comprises 18 items quantifying enjoyment and engagement in physical activity. The total score of the questionnaire was used to determine the level of satisfaction and kept for analysis.^23^ The PACES was completed at the first, seventh, and fifteenth class. To study the feasibility of implementing a leisure time physical activity, the CERT, a valid and reliable perceived exertion scale for children, was used at the end of each class to estimate the physical intensity of the dance exercise per class and for the whole dance program.^24^ A moderate physical intensity was targeted through the program (equivalent of 3–6 on the CERT), as exercise and physical activity recommendations for children with CP promote moderate physical intensity between one and three times per week.^2^ Both questionnaires were completed online, allowing non-verbal participants to write their answers. For those unable to type, communication was facilitated through yes or no responses, with the questionnaires administered by a parent.

### Data analysis

This study followed a mixed-method parallel design^18^ in which both quantitative and qualitative data were collected during the same phase and analyzed independently. The qualitative data were analyzed according to an inductive thematic analysis,^25,26^ which is a systematic procedure for analyzing qualitative data in which the analysis is guided by specific objectives. The primary purpose of this approach is to allow research findings to emerge from the dominant themes inherent in raw data, without the restraints imposed by structured methodologies.^27^ Following the initial reading of all anonymized interview transcripts (involving both dance instructors and participants), A.P-L. and M.H developed a codebook based on notes taken during the reading and emerging from the content of the transcripts. Sentences served as the coding units with text segments labeled with corresponding codes. Subsequently, one transcript was independently coded by A.P-L. and M.H. The coded transcript was then revised collaboratively to reach inter-rater agreement. Through an iterative process, the list of codes was then revised and finalized. All the transcripts were coded by A.P-L. and M.H. Upon completion of the coding phase, A.P-L. revised all the units of analysis for each code and synthesized the material into themes. For validation purposes, M.H. validated the synthesis. Following the coding, categories and themes were identified and presented to the research team to discuss the interpretation of the results. To minimize bias in the analysis, an iterative process of refining and redefining codes was followed to ensure accurate representation of the data. Additionally, the data analysis was validated by a second researcher to mitigate subjective biases. As the interviews were conducted in French, the verbatims illustrating the results of this article were translated into English by a bilingual team member (M.T.R.). The analysis was carried out using NVivo software version 1.7.1 (Lumivero, Denver, USA). Descriptive analyses are presented for the participation and retention rate as well as the results of the PACES and the CERT.

## Results

All participants completed the five-week dance program, giving a 100% retention rate. Through the program, the average participation rate was 86.7% ± 10.7%.

### Questionnaires

**Figure 1.**
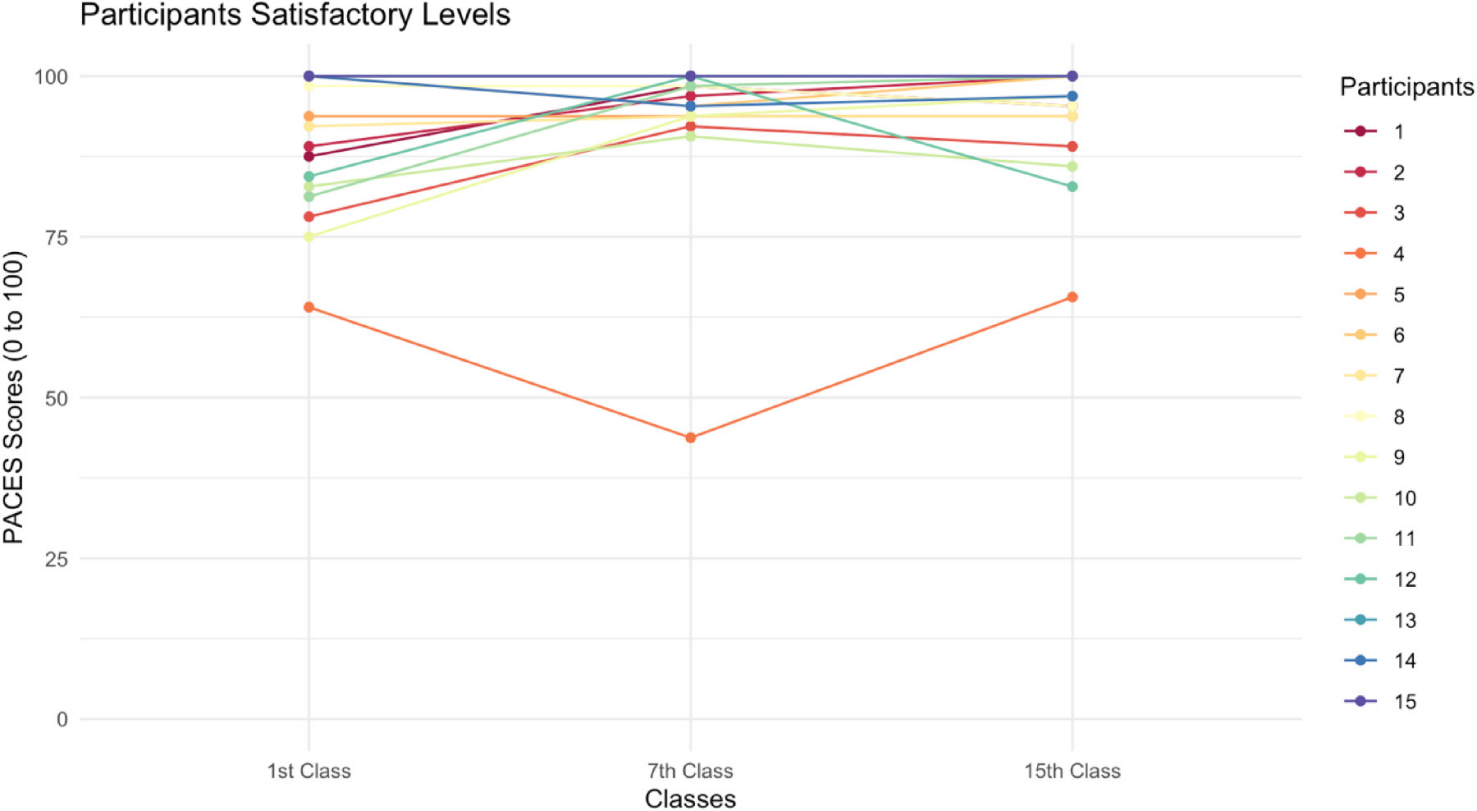
The participants’ satisfaction levels measured with the Physical Activity Enjoyment Scale (PACES) questionnaire. The questionnaire was filled out at the first, seventh and fifteenth classes (x-axis). Each participant is represented by a different line, and the PACES scores are on the y-axis. The value of every answer per participant is represented by a dot.

**Figure 2.**
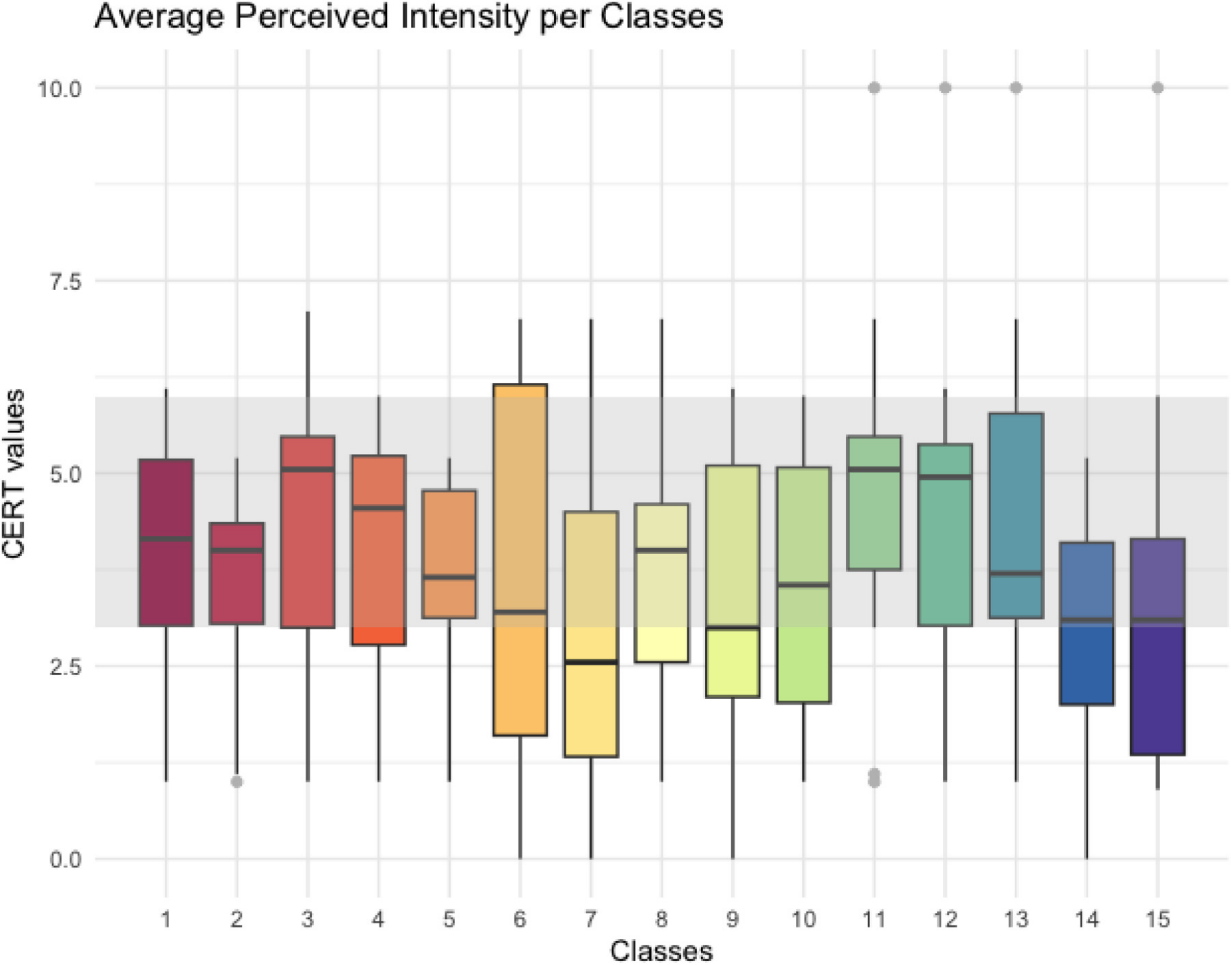
Average perceived intensity measured by the Children's Effort Rating Table (CERT). Each class is represented on the x-axis and the CERT value on the y-axis. The grey area represents the targeted intensity, i.e., moderate. Horizontal bars in the boxplot represent the median response. The lower and upper hinges correspond to the first and third quartiles (25^th^ and 75^th^ percentiles, respectively). Outliers are represented by gray dots.

The PACES average score was 88.4% ± 10.9, 92.7% ± 13.9, and 93.0% ± 9.3 for the first, the seventh and the fifteenth class, respectively. The results of the PACES per participant are presented in Figure 1. The average CERT score was 3.7 ± 1.3 throughout the dance program. Boxplots of the intensity perceptions measured by the CERT for each class are presented in Figure 2.

### Interviews

**Figure 3.**
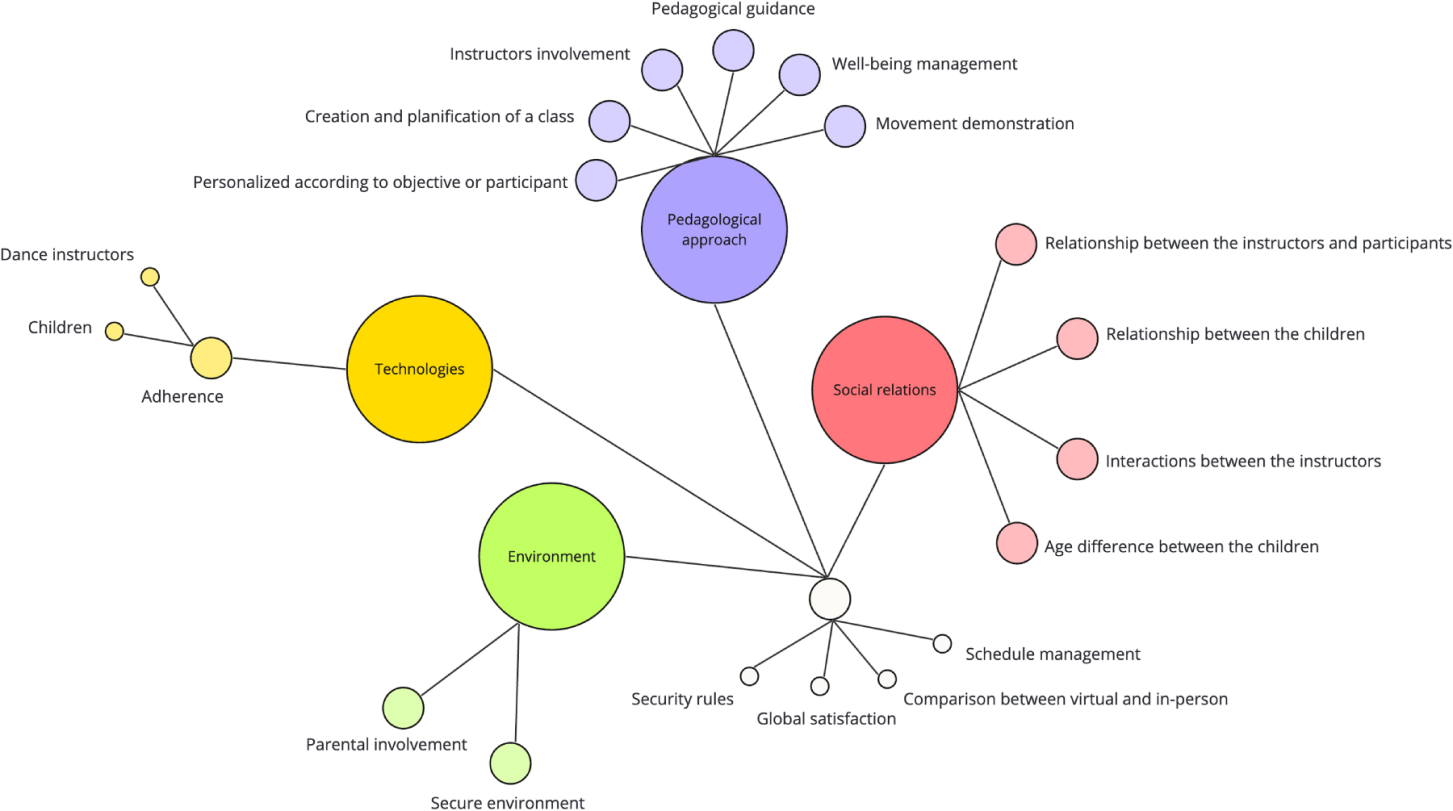
The conceptual map illustrates emerging themes and codes from the inductive thematic analysis. Each theme is represented by a larger colored circle while smaller circles represent individual code from the codebook. Lines indicate the relationship between codes and themes.

All dance instructors and young individuals with CP were present and participated in the interviews pre- and post-intervention. Data saturation was reached after the 3^rd^ and 7^th^ interviews with the dance instructors and participants, respectively, as no new themes emerged thereafter. The qualitative analysis highlighted four themes reflecting important elements to consider when implementing a teledance program in youth with CP. Each theme is represented by an illustrative quote from the participants to avoid redundancy, as similar views were expressed by multiple participants. Non-verbal participants contributed to the analysis through alternative forms of communication, ensuring a comprehensive reflection of the dataset. The four themes were 1) the technology; 2) the pedagogical approach; 3) the participant's environment; and 4) the social relations within the group. All themes are visually represented in a conceptual map (Figure 3) which was developed based on the inductive thematic analysis of the qualitative data.

#### Theme 1: the technology

At first, dance instructors reported various problems related to the virtual platform, such as the visual display and sound, and with the internet connection. Instructor #1 stated, “*It is sure that there are small technical problems that you don't have during an in-person dance session. Connection problems, you can't see the image properly, I never know if the sound they hear is the right one.”* Young individuals participating in the dance program reported similar problems. For example, regarding the internet connection problems, Participant #13 said, “*There was even one time when I wasn’t able to connect at all. It said I had no internet; it wasn’t working.*” Consequently, a longer period of adjustment than anticipated was necessary for the instructors to ensure a smooth class, as stated by Instructor #2: “*I mean, it was still a lot of things to deal with technically and it's not things that I would have considered initially.”*

As the teledance program advanced, the dance instructors noticed a change in the use of the technology. Technological problems were still present, but they were less disruptive and took less time in the course schedule. Dance instructors learned how to deal with unexpected technical issues. Instructor #3 stated, “*And there were times when it cut off [the image] for this participant […] at the beginning, she would cut off during the sessions to say “Something is going on” […] little by little in the program, she intervened less […] she got a little used to this bug there.”* Solutions to these problems were identified and implemented, e.g., using an Ethernet cable when possible. Moreover, to reduce the burden of technological difficulties and to ensure trouble-free operation of technologies with participants, dance instructors highlighted one of the features of the virtual platform: the waiting room. Instructor #3 stated: “ *[…] since I have a waiting room in my video conference platform […] it allows me to see them arrive, and then take the time admitting them.”*

Lastly, for some young individuals and their parents, the use of technology required a period of adjustment to successfully participate and attend the classes, as reported by the dance instructors and supported by the participants. Instructor #1 stated, “*So the first time [the participant] was late and then the next few times, [the participant] was early. And [the mother] said to me, ‘Yes, this time we got there early.’ I think it required them to anticipate at least the first few times as well, in the organization.”* In addition, the teledance program required some families to adapt less optimal devices to use all the videoconference platform features. Participant #7 reported, “*Since we had a small screen, I couldn’t see everyone who was there*.”

#### Theme 2: the pedagogical approach

Dance instructors reported a difference in the use of verbal feedback in an online class in comparison to an in-person class. Overall, dance instructors reported that feedback in the context of an online class was less accurate and less oriented towards one individual. Moreover, feedback was often used to get participants’ attention rather than providing information about the movement, especially during the first few classes. Instructor #1 stated, “*Then they were more and more attentive, which allowed us to give more feedback on the quality of the movements or the performance. I felt that I gave more feedback on the dance in the second half than in the first half [of the dance program] […].”*

According to the dance instructors, personalized activities based on an individual skill were deemed as more challenging in a virtual class compared to an in-person class. Individualization of activities was particularly challenging based on the young individuals’ progression, especially during a group session. Instructor #3 stated, “*I think the moment I saw the most of what was going on, […] it was a private class [the other participants were late to the class] between [participant's code] and me […] that was the first time I could take the time to look at the motor level to see what it looked like when [participant's code] was moving […] I realized that in the rotations, [participant's code] was unable to extend his or her arm […] I would not have been able to realize that otherwise…”* Participant #4 made a similar observation: “*In an in-person class, the teacher can see your movement […] if it's not correct, [the teacher] can come and correct you, then show you.”* Moreover, non-verbal signs of participants’ fatigue were difficult to distinguish in the online classes as mentioned by Instructor #2: “*I find non-verbal communication more difficult in a virtual dance session. […] it's also harder to detect if a child is less comfortable on something, is it because they are bored?”*

Dance instructors also noted that the teledance program could potentially reduce young individuals’ motivation and attention. Nonetheless, instructors reported that the overall organization and structure of the teledance program had a positive impact on the participants’ motivation.

#### Theme 3: the participants’ environment

The environment theme can be divided in two subthemes: physical and social, representing two distinct environments in which the participants evolved.

### Physical environment

Concerns were expressed by the dance instructors in the pre-interviews about the safety of the participants, but no incidents were reported during the program. Instructors reported that the absence of incidents was due to their capacity to understand what they were able to do. Instructor #1 stated, “*From there, if they do what they are capable of doing, […] we start with what they are capable of doing.”*

Because young individuals were in their home, the choice of activities was limited by the room dimensions as Instructor #2 stated: “*We couldn’t afford to do big jumps or runs, things like that from what we usually do in a dance room.”* However, the possibility to practice at home allowed the participants to have easier access to the dance classes which was highlighted as an advantage, as reported by Instructor #1: “*Some of them went on vacation, […] some of them connected from the campsite […] they were with their families, I don’t know where […].”* This convenience of attending the dance classes from home was also appreciated by the participants, as highlighted by Participant #14: “*With a virtual dance class […] It's because I stay at home so […] If I don’t have anything right after… I don’t have to wait too long because I just have to go upstairs! And I can have my food!”* However, reorganization of the family's schedule was also required to allow the participants to practice in a safe environment.

### Social environment

The dance instructors noted the presence and involvement of the participants’ parents. In some cases, the parents acted in support of the instructor when technological interruptions occurred or when the instructions were not understood by the child. For example, some parents provided additional instructions. Instructor #1 stated, “*I would hear her [the mother] and then she would rephrase the exercises to the participant and then sometimes she would go and help the participant with certain movements.”* Families’ presence or engagement during the teledance program was reported to be a source of motivation for the young individuals or a source of distraction. Instructor #2 stated, “*[Participant's code] was in open space and […] it was more complicated because [participant] was in the living room and then it was the place of passage in the house.”* The presence of a sibling bothered Participant #10: “*Yeah, because my brother, every time I dance, he turns on the TV, he gets in the way (annoyed). And that's it.”*

#### Theme 4: the social relations

Dance instructors reported a longer period to develop a collective sense with the participants in comparison to an in-person class. Nonetheless, dance instructors were surprised they were still able to develop a solid connection with the participants. Instructor #3 stated, “*And since we were on the phone a lot [to organize the dance schedule], we had a chance to talk to each other [the instructor and a participant]. […] I did not think I could build such an interesting bond with someone I met virtually then maybe never met in person there.”* Participants also echoed these sentiments, appreciating the social interactions facilitated by the program. Participant #15 noted, “*It's cool and I’m going to say the same reason as [Participant #13] because it allowed us to see other people and, on top of that, they were people from another continent too.”* Similarly, Participant #9 expressed, “*I think we had a lot of fun, you know, all of that. It was a pleasure to get to know other kids and other parents too, it feels good.”*

The presence of a group dynamic among all participants was noted by the instructors. For example, instructors were able to increase the chemistry of the group through non-verbal communication as stated by Instructor #1: “*they interacted maybe not with words, but they interacted through their movements. […] I realized that they knew the movements of the eight participants very well.”* Nonetheless, the teledance program was considered impersonal by the dance instructors since everyone could hear what other were saying. Instructor #3 stated, “*Already in an in-person class, sometimes there are participants who will stand side by side then they will go about their little lives sometimes, outside the group, so there are bonds that are created. […] And here, if they want to talk to each other, everyone hears, so it's sure that [um] … it's more complicated, I think.”* This more impersonal context was also perceived as an advantage for dance instructors as this required them to respond immediately as stated by Instructor #1: “*Someone speaks up and it's the only person who turns on their microphone and, they are heard, and the speaker is bound to respond.”*

On the other hand, a teledance program limited the implementation of activities targeting group interaction, as suggested by Instructor #2: “*In person, you can put them in teams, you can make them work together.”* However, according to the dance instructors, the collaboration between participants for the creation of choreography helped to improve the group dynamic. Instructor #1 stated, “*we composed around eight movements, each movement was the movement of a child. And, the very first class, when we learned the movements, we said, ‘Well … here, we do the bird of [code of a participant], we do the wave of [code of a participant]’. And so, although they had never met the other participants they would go, ‘Wednesday I’ll see [the code of a participant] or not this time?’ because they already knew each other since they had already heard the first name that had been circulated.”* The collaborative creation was an important aspect to encourage and stimulate interactions between the participants.

Overall, all the dancing instructors and a majority of the participating young individuals were satisfied with the teledance program. However, they all mentioned a preference for in-person class when given the choice between online and in-person classes. In summary, teledance seems to be a viable option when no other alternative is available. The integration of quantitative and qualitative results on global satisfaction is presented in Table 2.

**Table 2.** Joint display table for presenting congruent and discrepant findings on satisfaction.

Satisfaction	Congruent	Discrepant
PACES mean (SD): 91.4% (10.3)	P02: “We've done, uh, ballet, we've done, uh dance, jazz. It was good and I had a lot of fun"	P07: “Plus, doing virtual dance isn't so… it's not so much fun. Let's not lie to each other! I mean, you're all alone dancing like that… it's not as much fun as in person"
	P14: “Yes, I liked the experience, especially because I was able to dance with my sister … because she's been dancing for quite a long time"	I03: “She said you know, that for her, it was less interesting online… it wasn't even… it's not anger, it's not sad either. It's just you know, it's okay, it's not for everyone, and I don't do dance classes online either, because I don't like it, you know [laughs]."
	P15: “It's fun, and I'm going to say the same reason as P13, because it allowed us to see other people, plus people from another continent"	P09: “Of course, I would have preferred to dance in front of people who are really in front of me, in… in a real dance class"

Physical Activity Enjoyment Scale (PACES); SD (standard deviation); P (participant); I (dance instructor).

## Discussion

The aim of the study was to explore the perspectives of the dance instructors and participating children and adolescents on how to successfully implement an intensive teledance program for young individuals with CP using a mixed method design. Overall, the results suggest the feasibility of implementing an intensive teledance program that is perceived as enjoyable and creative. The findings highlight that factors such as technology, the pedagogical approach, the participant's environment, and the social interaction should be considered in the implementation of a teledance program. Other studies on online dance classes for children have investigated the effects of such interventions^28,29^; the present study contributes additional knowledge about the development and implementation of these interventions, enriching the existing literature.

A previous scoping review reported mixed evidence regarding children's compliance with online intervention.^30^ On one side, several studies have indicated reduced motivation levels in online classes,^16,31^ whereas others have reported high attendance rates.^28,31^ In particular, the present study reported high participation and a 100% retention rate, along with high satisfaction rate from the PACES, suggesting a real enjoyment among the participants. However, the qualitative data provided nuanced perspective. While some participants expressed satisfaction, citing the enjoyment and social aspect of interacting with others, including family, others expressed a preference for in-person classes, noting a diminished sense of fun and engagement when dancing physically alone, and a general lack of interaction. These results aligned with those of a previous study involving older adults, which reported that, although the online dance intervention was enjoyable and socially rewarding, participants preferred an in-person format.^31^

Maintaining motivation is a key ingredient reported in the literature to support participation and retention rates, particularly during long interventions.^32^ Motivation can be driven by interest, enjoyment, or social support.^32^ Accordingly, a few components of the dance program were designed with this in mind. Once a week, a combined class was held that brought together participants from both groups (France and Canada), allowing them to maintain a high level of satisfaction. This class enabled all participants to meet and increased group dynamics through the creation of a common choreography. The final class was structured as an online show for participants and their families, and the expectation of this event may have boosted participants’ motivation to complete the program. Moreover, the dance program was built around the participants’ goals, as well as dancing and music styles preferences,^15^ which could have enhanced their motivation to attend and complete the program. The flexibility of online classes to fit into the daily schedules of the participants and their families also likely influenced participation and retention rate. With online classes, participants could attend from wherever they wanted with access to at least a cellular or WiFi network and a smartphone. For instance, some participants continued attending the classes while traveling or on vacation. Based on the results of the present study, it can be concluded that the strategies employed to stimulate engagement and motivation were effective in this context, as evidenced by the high satisfaction and participation, which outweighed the potential drawbacks of online classes. Furthermore, incorporating instructors’ perspectives provided additional insights into the strategies employed. Strategies for increasing motivation and participation in online courses should be further investigated in future studies.

The average physical intensity perception during the teledance program was moderate, suggesting that the targeted intensity was reached, aligning with the physical activity recommendations for young individuals with CP.^2^ Nonetheless, some participants reported a light intensity of some classes. During the dance program, instructors reported difficulties in assessing non-verbal indicators of fatigue or the quality of movement. As reported in previous studies, online classes limit the direct observation of the child^16^ and personalized feedback.^31^ In this setting, it may be more difficult to target an intensity or to adjust the intensity of the activity to the participant's characteristics. Feedback is a tool for maintaining a high level of engagement and commitment to activities, which can facilitate targeted intensity of physical activity. However, dance instructors reported reduced oriented feedback during the online dance program and more feedback on the child's attention, which in turn could limit guidance towards the targeted intensity. Nonetheless, an online dance program with a reduced amount of feedback remains positive in a recreational context where the aims are oriented towards the child's participation and enjoyment. However, in a dance program with therapeutic goals, the type of feedback given in an online class might not be optimal,^33,34^ as the provision of extrinsic feedback may have an influence on the retention of motor learning.

Social interactions emerged as one of the important components of the dance class for instructors and participants. Both reported a lack of social interactions or the difficulty to develop a sense of community. In previous studies, contact with peers and informal interactions such as being together, playing, and laughing have been reported as lacking or missing in online classes.^17,35^ Moreover, the lack of social contact was previously stated as one of the main barriers to the uptake of tele-programs.^36^ In the present study, a high participation and retention rate was observed, despite the reported lack of social interaction. Dance has an inherent relational and social dimension compared to other leisure time physical activity.^37^ Therefore, it is crucial to consider the limits of social interaction and to develop strategies to enhance the relational and social dimensions during an online dance program. For example, in the present study, the collective creation of choreography was reported as beneficial to the relational and social dimensions by both instructors and participants. On the other hand, a recent study suggested combining in-person and online classes when possible, to build the relationship between participant and instructor in person first.^38^

Based on the perspectives of the dance instructors and participants, some recommendations can be made to ensure a successful implementation of an online dance program. As stated by Rugh et al.^19^ and Moo et al.,^38^ attention should be paid to technology. First, the video conference platform should be intuitive, easy to use, and customizable (e.g., alternating between a group view or an individual one). A period for customization of the platform for both the instructors and the participants and their families should be planned prior to the intervention.^38^ Second, personalization of the goals, choice of music, and dancing styles may increase satisfaction, motivation, and participation. Third, devoted time for social interaction should be integrated in the program. As a direct consequence, collaborative works within the dance program may increase relational and social aspects. Finally, as participants are in their family environment, dance instructors need to be aware of both the positive and negative repercussions of the presence of family members during the class. Dance instructors should take this into consideration when planning the intervention.

### Study limitations

This study, conducted during the lockdowns following the COVID-19 pandemic, has a few limitations. Firstly, the pandemic itself may have acted as a confounding factor, potentially impacting participants’ behavior and their environmental context. Secondly, the relatively small sample size may limit the generalizability of the findings. Nevertheless, the objective was to delve into the perspective of key stakeholders, and consequently, the results are directly tied to the study's context, with the main findings stemming from a qualitative analysis. Finally, when conducting interviews with children, their expressive abilities may be constrained, particularly in younger age groups, posing a challenge in fully capturing their perspective. The qualitative insights were complemented with quantitative data, adopting a mixed-method approach. Future studies should investigate the relationship between participants’ characteristics and their satisfaction, enjoyment, and participation. From an anecdotal standpoint, children with both higher and lower motor function appeared equally engaged, satisfied, and motivated to participate in the program.

## Conclusion

Programs encouraging participation in leisure time physical activity for children and adolescents with CP are important given their reduced level of physical activity in comparison to typically developing peers. Overall, the results suggest that a teledance program is a promising modality to increase participation in leisure time physical activity, as it is an enjoyable activity with a social and relational dimension, requiring minimal equipment and travel. The findings emphasize the importance to consider and provoke social interactions between participants and dance instructors to develop and cultivate a sense of community. Ultimately, greater participation in leisure time physical activity could help to achieve higher fitness levels, reduce secondary complications, and improve autonomy and overall quality of life.

## Supplemental Material

sj-docx-1-prm-10.1177_18758894251324317 - Supplemental material for Towards a successful teledance program for youth with cerebral palsy: A mixed-method study with the instructor’s perspective Supplemental material, sj-docx-1-prm-10.1177_18758894251324317 for Towards a successful teledance program for youth with cerebral palsy: A mixed-method study with the instructor’s perspective by Annie Pouliot-Laforte, Claire Cherrière, Margaux Hebinck, Jessica Tallet, Catherine Donskoff, Louis-Nicolas Veilleux, Martin Lemay and Maxime T Robert in Journal of Pediatric Rehabilitation Medicine
