# Supplementary material for: Towards a successful teledance program for youth with cerebral palsy: A mixed-method study with the instructor’s perspective
Source: J Pediatr Rehabil Med. 2025 Apr 10:18758894251324317. Online ahead of print. doi: 10.1177/18758894251324317 (PMC13292708; doi:10.1177/18758894251324317)
Supplement: Supplementary file 1 [file sj-docx-1-prm-10.1177_18758894251324317.docx]

**Supplementary Data**

**Interview questions**

*Pre- intervention*

1. Have you ever tried dancing classe? Tell me about it.

2. What's your experience with taking classes online? What was it like?

3. How do you feel about using online plateforms to learn or exercise?

4. Imagine there's a program where you can learn dance online. How does that make you feel?

5. What would make you super excited about learning dance on a computer or tablet?

6. What are your personal objectives that you wish to accomplish through an online dance program?

*Post- intervention*

1. Tell me about what happens in your dance classes.

2. What feelings do you get from attending your dance classes?

3. Share a story where something really good happened during your dance lessons.

4. Think of a moment during your dance classes you'd change if you could. What is it?

5. Have you noticed anything different when you take dance classes online compared to when you're all dancing together? What's that like?

6. How do you feel about the dance program overall? Are there things you're happy with or things you wish were different?

7. In what ways did the dance classes help you achieve your goals, or how did they fall short?

**The questions above were formulated for the participants. The questions asked to the dance instructors were adapted based on these questions.*
