# Supplementary material for: Towards a successful teledance program for youth with cerebral palsy: A mixed-method study with the instructor’s perspective
Source: J Pediatr Rehabil Med. 2025 Apr 10;18(2):120–31. doi: 10.1177/18758894251324317 (PMC13292708; doi:10.1177/18758894251324317)
Supplement: sj-docx-1-prm-10.1177_18758894251324317 - Supplemental material for Towards a successful teledance program for youth with cerebral palsy: A mixed-method study with the instructor’s perspective [file sj-docx-1-prm-10.1177_18758894251324317.docx]

**Supplementary Data**

| **Interview questions** |  |
| --- | --- |
| *Pre- intervention* |  |
| 1. Have you ever tried dancing classe? Tell me about it. |  |
| 2. What's your experience with taking classes online? What was it like? |  |
| 3. How do you feel about using online plateforms to learn or exercise? |  |
| 4. Imagine there's a program where you can learn dance online. How does that make you feel? |  |
| 5. What would make you super excited about learning dance on a computer or tablet? |  |
| 6. What are your personal objectives that you wish to accomplish through an online dance program? |  |
|  |  |
| *Post- intervention* |  |
| 1. Tell me about what happens in your dance classes. |  |
| 2. What feelings do you get from attending your dance classes? |  |
| 3. Share a story where something really good happened during your dance lessons. |  |
| 4. Think of a moment during your dance classes you'd change if you could. What is it? |  |
| 5. Have you noticed anything different when you take dance classes online compared to when you're all dancing together? What's that like? |  |
| 6. How do you feel about the dance program overall? Are there things you're happy with or things you wish were different? |  |
| 7. In what ways did the dance classes help you achieve your goals, or how did they fall short? |  |
| **The questions above were formulated for the participants. The questions asked to the dance instructors were adapted based on these questions.* |  |
|  |  |
|  |  |
